# The Spectrum of Autoantibodies in Adult Patients With Idiopathic Pulmonary Hemosiderosis: A Brief Review of the Literature

**DOI:** 10.7759/cureus.24169

**Published:** 2022-04-15

**Authors:** Biplab K Saha, Alyssa Bonnier, Santu Saha, Baidya N Saha, Nils T Milman

**Affiliations:** 1 Pulmonary and Critical Care Medicine, Ozarks Medical Center, West Plains, USA; 2 Critical Care, Goldfarb School of Nursing at Barnes Jewish College, Saint Louis, USA; 3 Internal Medicine, Saha Clinic, Narail, BGD; 4 Department of Clinical Biochemistry, University College Zealand, Næstved, DNK

**Keywords:** rheumatoid factor, anca, ana, autoantibody, adult, idiopathic pulmonary hemosiderosis

## Abstract

While autoimmune antibodies or autoantibodies have been reported sporadically in adult patients with idiopathic pulmonary hemosiderosis (IPH), their true prevalence is unknown. The question as to whether any difference exists between antibody-positive and negative patients has not been explored. The primary objective of this paper was to assess the spectrum of autoantibody testing and its positivity rate. The other objectives included a comparative analysis of demographics, symptom onset, clinical manifestations, and differences in clinical outcomes between antibody-positive (cohort A) and negative (cohort B) patients. To that end, we conducted a retrospective review of the relevant published literature. Multiple databases were searched to retrieve studies published between 1990 and 2022.

A total of 35 studies, involving 38 patients, were identified. Five of these patients had a positive autoantibody. Patients in cohort A were older and more likely to be male. The frequencies of testing for these antibodies were as follows: antineutrophil cytoplasmic antibody (ANCA): 37/38 (97.4%), antinuclear antibody (ANA): 31/38 (81.6%), and anti-glomerular basement membrane antibody (anti-GBM): 30/38 (78.9%); 5/38 (13.2%) patients tested positive for an autoantibody, and two of these patients were positive for ANA, two for antithyroid antibody, and one patient tested positive for ANCA, rheumatoid factor (RF), and granulocyte monocyte-colony stimulating factor (GM-CSF) antibody. There was no difference between the cohorts regarding their clinical presentations, recurrence risks, and survival.

The occurrence of autoantibodies is uncommon in adult IPH patients. This is in contrast with the pediatric IPH patient population, where the prevalence is much higher (26.4% vs. 13.2%), and the antibodies are more diverse. Unlike pediatric patients, adult patients with autoantibodies do not necessarily have worse outcomes.

## Introduction and background

Idiopathic pulmonary hemosiderosis (IPH) is a rare cause of diffuse alveolar hemorrhage (DAH) in adults. As there are no specific clinical, laboratory, or radiologic findings that distinguish IPH from other causes of DAH, any patient suspected to have DAH typically undergoes a thorough investigation to rule out other more common causes of alveolar hemorrhage, such as vasculitides [1,2], post-capillary pulmonary venous congestion [3,4], connective tissue diseases [5], infection [6], exposure to inhalational toxins and drugs [7], and hematologic diseases [8]. Clinicians often perform an extensive serologic workup before resorting to lung biopsy for a definitive diagnosis of IPH [1,9,10].

Despite definite proof, IPH is considered an immune-mediated disease, and hence the name immune-mediated pulmonary hemosiderosis (ImPH) has been proposed for it [5,11]. One of the critical aspects of such considerations is the presence of autoantibodies in patients with IPH. Although autoantibodies have been reported in pediatric IPH patients consistently [12], the true prevalence of autoantibodies in adults has been largely unknown until recently. We have recently discussed the prevalence of autoantibodies in a cohort of adult IPH patients as reported in the literature [13]. Similar to pediatric patients, a majority of these patients had antibodies suggestive of celiac disease (CD), a combination known as Lane Hamilton syndrome (LHS) [12,14,15].

Several studies involving pediatric patients with IPH have demonstrated worse outcomes in patients with autoantibodies [16,17], but no such data exists for adult patients. In light of this, we have undertaken this review to provide a detailed analysis of the type of antibodies that have been tested for patients with IPH, their positivity rate and differences in demographics, clinical presentations, and outcomes in patients with or without autoantibodies. We have excluded patients with CD for this paper unless these patients also tested positive for other autoantibodies.

## Review

Materials and methods

This was a retrospective review of the previously reported cases of adult IPH patients. The Medline, Embase, and PubMed databases were searched using the following keywords to identify appropriate citations for this manuscript: "Idiopathic pulmonary hemosiderosis OR IPH AND adults"; "idiopathic pulmonary hemosiderosis OR IPH AND antibody"; "idiopathic pulmonary hemosiderosis OR IPH AND autoantibody"; and "idiopathic pulmonary hemosiderosis OR IPH AND autoimmune."

Inclusion criteria

The following manuscripts were included in our review: (1) Studies involving patients who were diagnosed with IPH as an adult (aged 18 years or above) and tested for autoantibodies during evaluation; (2) Studies in which the diagnosis of IPH was made with consistent clinical and radiologic findings and demonstration of hemosiderin-laden macrophages (HLM) from respiratory tract samples, obtained either by bronchoalveolar lavage or spontaneous expectoration (sputum), and/or lung biopsy consistent with IPH; (3) Articles published in the English language in peer-reviewed journals between January 1, 1991, and February 15, 2022.

Exclusion criteria

The exclusion criteria were as follows: (1) Articles where the patient was diagnosed with IPH as a child, even if the patient was reported as an adult; (2) Studies on pediatric patients with IPH and autoantibodies; (3) Articles on patients with serologic LHS as this has been reported separately unless the patient also had non-CD antibody; (4) Meeting abstracts; (5) Articles in a non-English language.

Patient cohorts

The patients included in this review were divided into two cohorts: (1) Cohort A: patients who were tested for autoantibodies and were positive for one or more autoantibodies; (2) Cohort B: patients who were tested but returned a negative screen.

Study objectives

The primary objectives of the study were to report the pattern of autoantibody testing in the reported adult patients with IPH and their rate of positivity. The secondary objective was a comparative analysis of demographics, symptom onset, clinical manifestations, and differences in clinical outcomes between the cohorts.

Statistical analysis

The descriptive and inferential statistical analyses were performed using the SPSS Statistics version 28 (IBM, Armonk, NY). The interval data were reported as mean and standard deviation (SD) and median and interquartile range (IQR). The association between categorical data was assessed by chi-square or the Fisher’s exact test. The difference between interval data was analyzed by an independent t-test.

Results

Study Characteristics and Patient Demographics

**Figure 1:**
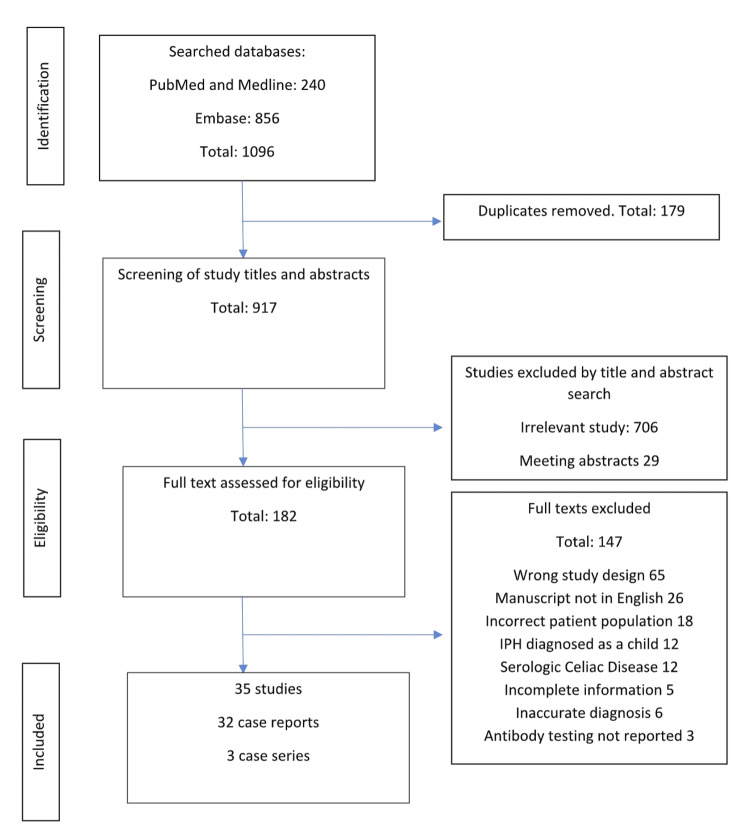
Flow chart showing the study selection process IPH: idiopathic pulmonary hemosiderosis

A total of 35 studies fulfilling the inclusion criteria were identified; 32 of them were single cases and three were case series with two patients in each. The study selection process is illustrated in Figure 1.

Five patients were positive for autoantibodies (cohort A) [18-22], and the rest were tested but turned out to be negative for any antibodies (cohort B). The mean (SD) and median (IQR) ages at diagnosis of IPH for cohort A were 58.2 (19.9) years and 50 (36.5) years, respectively. The mean and median ages for cohort B were 35.15 (15.68) years and 29 (27.5) years, respectively. Patients in cohort A were older than cohort B at the time of IPH diagnosis but the difference was not statistically significant (two-sided independent sample t-test, p=0.058). Of note, four out of five (80%) patients in cohort A were males compared to 18/33 (54.5%) in cohort B.

Testing for Antibodies

The most commonly tested antibodies were antineutrophil cytoplasmic antibody (ANCA), antinuclear antibody (ANA), and anti-glomerular basement membrane (anti-GBM) antibodies. The frequencies of testing for these antibodies were as follows: ANCA: 37/38 (97.4%), ANA: 31/38 (81.6%), and anti-GBM: 30/38 (78.9%). The other autoantibodies that were tested with a lower frequencies included rheumatoid factor (RF): 16/38 (42.1%), anti-double-stranded DNA (anti-dsDNA): 10/38 (26.3%), antiphospholipid antibody (APLA): 9/38 (23.7%), anti-cyclic citrullinated peptide (anti-CCP): 4/38 (10.5%), ribonuclear protein (RNP): 3/38 (7.9%), Scl70: 2/38 (5.3%), smooth muscle antibody (SMA): 2/38 (5.3%), anti-thyroid antibody: 2/38 (5.3%), anti-mitochondrial: 1/38 (2.6%), and anti-granulocyte monocyte colony stimulating factor (anti-GM-CSF) antibody: 1/38 (2.6%).

Prevalence of Autoantibodies

Of note, 5/38 (13.2%) patients tested positive for an autoantibody. Two of these patients were positive for ANA [20,21], two for antithyroid antibody [19,21], and one patient each tested positive for ANCA [21], RF [18], and GM-CSF antibody [22]. Two of these five patients also tested positive for CD antibodies [20,21]. One patient had positive ANA, tissue transglutaminase (TTG), anti-thyroid antibody, as well as ANCA positivity [21].

Differences in Clinical Presentation Between Cohorts

A comparative analysis of clinical presentations by chi-square or Fisher’s exact test revealed no statistical difference in the incidence of hemoptysis (80% vs. 66.67%), cough without hemoptysis (20% vs. 36.3%), dyspnea (80% vs. 87.5%), chest pain (0% vs 9%), anemia (75% vs. 87.8%), respiratory failure (0% vs. 18.1%), or systemic symptoms (40% vs. 36.3%) between the cohorts. Similarly, there was no statistical difference in the number of patients with the classic triad (40% vs. 72.7%).

Comparison of Disease Recurrence and Survival Between Cohorts

The recurrence rates were 50% and 61.5% for cohorts A and B, respectively. The survival rates were 100% and 78.3% at the time of final follow-up in cohorts A and B, respectively. The differences in recurrence or survival between the cohorts were not statistically significant as per the chi-square test.

Discussion

To our knowledge, this is the only paper that has summarized the autoimmune workup performed for adult patients with IPH and engaged in a comparative analysis of patients with or without autoantibodies in terms of demographics, clinical manifestations, and outcomes. We have identified several important findings in this review. Firstly, the reported workup for IPH patients was highly variable. The number of autoantibody testing in individual patients varied significantly. As expected, nearly all patients had been evaluated for ANCA antibodies, but testing for other autoantibodies had been limited. Second, the prevalence of positive autoantibody was significantly lower compared to pediatric patients. Third, the diversity of autoantibodies was also less than in pediatric patients. Fourth, the patients with a positive autoantibody tended to be older and male, but there was no difference in clinical presentations and outcomes between the cohorts.

IPH is a rare cause of DAH, without any known pathophysiology [8]. Several hypotheses have been proposed to describe the pathobiology of IPH, but the exact mechanism is still unknown [1,8]. We have recently proposed a new hypothesis for the occurrence of DAH in IPH, which explains not only the clinical and histopathologic findings but also the association with CD and the positive response to a gluten-free diet (GFD) [23]. We believe that the autoantibodies in these patients are not pathogenic, but an expression of overall propensity towards immune dysregulation. Consistent with the concept of autoimmune dysregulation was the presence of multiple autoantibodies in several of our patients. Stainer et al. have reported the coexistence of ANA, ANCA, CD, and anti-thyroid antibodies in the same patient [21]. Similarly, the patient reported by Leaker et al. had CD in addition to ANCA-associated vasculitis (AAV) [24]. Although the coexistence of two rare diseases, like IPH and AAV, could be coincidental, there is likely a unifying mechanism, as proposed in our hypothesis.

The search for ANCA is often the most crucial step in the evaluation of patients with DAH, as the management and prognosis of AAV differ significantly from other causes of DAH. Interestingly, the identification of ANCA did not occur until the mid-1980s, and it was not routinely checked till the 1990s [25]. Therefore, in this manuscript, we only included patients reported after 1990. As expected, all but one patient were evaluated for ANCA antibodies. We identified only one patient diagnosed with IPH as an adult who developed ANCA during further follow-up [21]. It is noteworthy that there are other reports of ANCA development in IPH patients as an adult, but in all of these instances, the diagnosis of IPH was made when they were children [21,24,26]. A thorough description of these cases is provided in Table 1. All but one of these patients [24] were diagnosed with IPH with lung biopsies and developed ANCA after many years (range: 8-18 years). As seropositivity for anti-proteinase 3 (anti-PR3) antibody is associated with worse outcomes in patients with AAV, some authors have proposed a classification system based on serology rather than classical phenotypic differentiation into granulomatosis with polyangiitis (GPA), microscopic polyangiitis (MPA), and eosinophilic granulomatosis with polyangiitis (EGPA) [27]. Intriguingly, all of the patients we identified were positive for anti-myeloperoxidase (anti-MPO) antibody and primarily with a perinuclear (pANCA) distribution on ELISA. Therefore, it is possible that at least some of the patients diagnosed with IPH may have suffered from an attenuated form of AAV. Unfortunately, none of these patients underwent a repeat lung biopsy after developing ANCA to definitively demonstrate histopathologic evidence of vasculitis but were instead treated with enhanced immunosuppression based on the clinical information.

**Table 1:** Reported cases of IPH with ANCA positivity ANA: antinuclear antibody; ANCA: antineutrophil cytoplasmic antibody; APLA: antiphospholipid antibody; AZA: azathioprine; cANCA: cytoplasmic ANCA; CD: celiac disease; CS: corticosteroid; CT: computed tomography; CYC: cyclophosphamide; GBM: glomerular basement membrane; HCQ: hydroxychloroquine; HLM: hemosiderin-laden macrophages; IPH: idiopathic pulmonary hemosiderosis; MMF: mycophenolate; MPO: myeloperoxidase; MTX: methotrexate; NS: not specified; pANCA: perinuclear ANCA; PR3: proteinase 3; PFT: pulmonary function test; TTG: tissue transglutaminase

Author	Age at the time of reporting (year), gender	Presenting symptoms	Duration of presenting symptoms	Age at IPH diagnosis (years)	Autoantibody tested/positive antibodies	Positive antibody after IPH diagnosis (years)	Lung histopathology		Initial treatment	Recurrence of IPH		Clinical course		Follow-up (years)	Respiratory outcomes	Other organ involvement
Stainer et al., 2019 [21]	32, F	Cough, hemoptysis, exertional dyspnea since 14 years of age	18 years	14, workup not specified except bronchoscopy and surgical lung biopsy	Autoimmune screening at 14 years of age (NS). At 21 years of age, autoimmune screening (NS) revealed atypical ANCA positivity on IIF Anti-MPO, anti-PR3: negative, pANCA, anti-MPO 27U/ml	18	HLM in the alveolar space, mild bronchiolitis with lymphoid aggregates, type 2 pneumocyte hyperplasia, and mild chronic interstitial inflammation (age 14)		HCQ at age 14. Over the years, she received IV CS, AZA, MTX, and MMF. At age 32, she received IV CCP and rituximab	Yes, between ages 14 and 32		No hemoptysis after IV. CYP and rituximab		0.83 year after initiation of CYP and rituximab	Improved shortness of breath	Renal impairment with hematuria
Stainer et al., 2019 [21]	42, M	Presented at 34 years of age with hemoptysis, exertional dyspnea, anemia requiring blood transfusion	2 years before presenting at 34 years of age	34	Autoimmune workup (NS): negative. Speckled ANA, pANCA, anti-MPO 14U/ml, anti-thyroid, anti-TTG antibodies (duodenal biopsy: normal)	8	HLM in the alveolus, perivascular lymphoid aggregate and noncaseating granuloma, hemosiderin deposition on the elastic fiber of pulmonary artery (age 34)		Systemic CS	Recurrence of hemoptysis after 6 years after steroid tapering. The patients also had arthralgia		MTX was added to CS with an improvement of symptoms but persistent hemoptysis		At least 2 years after the autoantibody was identified	Worsening chest CT and PFT	None
Stainer et al., 2019 [21]	19, M	Evaluation for ILD at 16 years of age with a known history of IPH	Ongoing hemoptysis despite immunosuppression from 7 years of age; respiratory failure at 11 years of age requiring ventilation; lung biopsy performed	7	Autoimmune workup (NS): negative at 11 years of age, pANCA, anti-MPO 4.6U/ml	12	Hemosiderin-laden macrophages without any evidence of vasculitis or granuloma. Type 2 pneumocyte hyperplasia and small lymphoid aggregates		Intermittent courses of CS initially. The patient was on CS, HCQ, and azithromycin after the episode of respiratory failure at age 11	Recurrent hemoptysis		Azathioprine was added to CS, HCQ, and azithromycin after anti-MPO identified at the age of 19		NS	Stable symptoms and PFT	None
Freitas et al., 2015 [26]	21, F	New-onset polyarthralgia in a patient with open biopsy-proven IPH at the age of 14	12 years	4, with a negative serologic workup and without a lung biopsy	ANA, anti-GBM, ANCA, APLA, CD, milk protein: negative, cANCA 1:640 MPO 101 U/ml	12	Extensive recent and prior alveolar hemorrhage, septal thickening, nonspecific inflammation, elastic fiber disruption without any granuloma or vasculitis (age 14)		CS and HCQ later switched to AZA due to side effects at age 12. Rituximab was started after 2 doses of CYC after the identification of ANCA at the age of 16	None		The patient was asymptomatic at age 21. Reduction in ANCA and MPO titer		5 years	No limitation in finishing nursing school	None
Leaker et al., 1992 [24]	22, M	Recurrent hemoptysis	8 years	14	Anti-GBM antibody: negative, ANCA 1:80 Anti-MPO 1:80. The patient was diagnosed with CD from jejunal biopsy at age 14	8	Not performed	CS and CYS. Later switched to CS and AZA		None	The ANCA antibody turned negative		NS		NS	Segmental necrotizing glomerulonephritis

Other autoantibodies identified in our review were ANA [20,21], RF [18], anti-thyroid [19,21], and anti-GM-CSF antibodies [22]. In addition, we have identified several additional patients with autoantibodies reported as adults after being diagnosed with IPH as children (Table 2) [28-31]. ANA is a common autoantibody with a prevalence of approximately 2.5% in the healthy general population [32]. We found a prevalence of 5% among our patients, which is not very different from the overall prevalence. Moreover, the titer of the ANA is also crucial, as a titer of 1:80 is often considered a positive screen. However, using a cut-off value of 1:40 would significantly increase the prevalence of ANA. The ANA titer was 1:160 in the study by Nishino et al. [20] and was reported to be mildly positive by Stainer et al. [21].

**Table 2:** Cases of positive autoantibodies in adult patients with idiopathic pulmonary hemosiderosis (IPH) ANA: antinuclear antibody; ANCA: antineutrophil cytoplasmic antibody; AZA: azathioprine; cANCA: cytoplasmic ANCA; CCP: cyclic citrullinated polypeptide; CS: corticosteroid; dsDNA: double-stranded DNA; GBM: glomerular basement membrane; HLM: hemosiderin-laden macrophages; IPH: idiopathic pulmonary hemosiderosis; MPO: myeloperoxidase; NSIP: nonspecific interstitial pneumonia; pANCA: perinuclear ANCA; PR3: proteinase 3; RF: rheumatoid factor; SSA: Sjogren syndrome antibody A; SSB: Sjogren syndrome antibody B; TG: thyroglobulin; TPO: thyroid peroxidase; TTG: tissue transglutaminase

Author	Age (year), gender	Presenting symptoms	Duration of presenting symptoms	Age at IPH diagnosis (years)	Autoantibody tested/ positive antibody	Positive antibody after IPH diagnosis (years)	Lung histopathology	Initial treatment	Recurrence of IPH	Clinical course	Follow-up (years)	Respiratory outcomes	Other organ involvement
Walsh et al., 2021 [22]	50, M	Worsening dyspnea and hemoptysis	6 weeks	50	NS, GM-CSF 53.3 microgm/ml	At diagnosis	Fresh alveolar hemorrhage, HLM in the alveolar space. Also found to have PAP	CS	None	The patient remains stable with tapering of CS	NS	Largely normal PFT with mild reduction of DLCO	None
Ren et al., 2020 [28]	21, F	Confirmation of suspected IPH	Shortness of breath for 2 years. Arthralgias and polyarticular joint swellings for 2 months; no hemoptysis	6, inadequate information regarding workup	ANA, ANCA, anti-dsDNA, anti-Scl-70: negative, anti-CCP 466U/ml (normal <25U/ml), RF 1710IU/ml (normal <20IU/ml)	15	Hemosiderin-laden macrophages without any evidence of vasculitis, organizing alveolitis, or granulomatosis. Interlobular septal thickening due to fibrosis	CS	None	Normalization over 8 months	0.67 year	Improved chest radiology. Stable to mildly improved PFT	None
Yanagihara et al., 2018 [29]	32, M	Dry cough, exertional dyspnea	1.5 years	7	ANA, ANCA, anti-CCP, RF, anti-dsDNA, anti-smith, RNP, anti-Scl-70, anti-Jo, anti-GBM, anti-MPO, anti-PR3, SSB: negative, ANA 1:320 anti-SSA 240U/ml	25	Chronic inflammatory infiltrates, lymphoid aggregates, and interstitial fibrosis consistent with NSIP. Iron deposition in the elastic fibers of the vessels. No vasculitis or granulomatosis	The patient was on CS from age 7 to 18	None	The patient was started on CS again after the diagnosis of SS at 34	NS	Near-complete resolution of symptoms	Lacrimal gland
Dutkiewicz et al., 2010 [18]	74, M	Hemoptysis, exertional dyspnea, loss of appetite, weight loss	2 years	74	ANA, ANCA, anti-CCP, anti-dsDNA, anti-phospholipid panel, anti-GBM, anti-MPO, anti-PR3, SSA, SSB: negative, RF 315	At diagnosis	Evidence of recent and past intraalveolar hemorrhage and arterial mural hypertrophy. No vasculitis, granulomatosis, or thrombosis	CS	Yes	Recurrent hemoptysis with reduction of steroid dose even after the addition of azathioprine	0.67 year	NS	None
Fuji et al., 2010 [19]	83, M	Hemoptysis shortly after thyroidectomy	NS	83	ANA, ANCA, anti-dsDNA, anti-GBM, anti-MPO, anti-PR3: negative, anti-TPO anti-TG	At diagnosis	Not performed	CS	None	Improved clinically and radiologically	NS	The patient was reported to be asymptomatic	None
Nishino et al., 2010 [20]	50, F	Fatigue, marked exertional dyspnea, cough, and hemoptysis	NS	50	ANCA, anti-dsDNA, anti-GBM: negative, ANA 1:160 anti-endomysial, and TTG: positive	At diagnosis. The patient was diagnosed with CD 10 years ago	Intraalveolar HLM	NS	NS	NS	NS	NS	None
Louie et al., 1993 [30]	19, F	Hemoptysis, progressive dyspnea	Diagnosed with IPH at 3 years of age, recurrent episodes of hemoptysis and respiratory failure	3	ANA, anti-dsDNA, anti-GBM, SSA: negative, RF 1:1280	15	At age 3, diffuse intraalveolar HLM. Pulmonary fibrosis and no evidence of vasculitis, granulomatosis, or organizing alveolitis	CS	Yes	Recurrent hemoptysis and respiratory failure	19 years	Oxygen-dependent respiratory failure but control of life-threatening hemorrhage with high-dose steroid	Rheumatoid nodule at the right elbow
Lemley et al., 1986 [31]	21, M	Diagnosed with IPH at 11 years of age. Presented at 21 years of age with polyarticular arthritis, no hemoptysis or new respiratory symptoms	From 7 years of age	11	None, RF 1:1024	10	At age 11, HLM in the alveolus, fibrosis of alveolar septa. No vasculitis, immunoglobulin, or complement deposition	AZA	None specified	Improved radiologically and symptomatically	10 years	No recurrence of pulmonary hemorrhage and AZA discontinued after 3 years	Polyarthritis

RF is a nonspecific antibody. In addition to patients with rheumatoid arthritis, RF can be present in healthy individuals and patients with chronic infection and malignancy [33,34]. Rheumatoid arthritis is the most common autoimmune disease and can cause DAH due to vasculitis [35]. All patients in this review (Table 2) who had RF and were diagnosed with concomitant RA had no histopathologic evidence of vasculitis on lung biopsy [18,28,30,31]. Therefore, the coexistence of IPH and RA could suggest immune dysregulation in patients with IPH rather than causation. Similarly, the anti-thyroid antibodies [19] and coexistence of IPH with pulmonary alveolar proteinosis [22] are likely to represent the same phenomenon.

We have previously reported on the prevalence of autoantibodies in children [12]. In our review, the overall prevalence of autoantibodies was 26.4%. Compared to that, the prevalence in adults was much less common and less diverse (ANA: 5.3% vs. 20.3%; ANCA: 2.6% vs. 17%; RF: 2.6% vs. 12%; ant-dsDNA: 0% vs.9.1%; and SMA: 23.2% vs. 0%). None of the adult patients were positive for SMA or anti-dsDNA. One of the reasons for the discrepancies could be that adults were not evaluated for a diverse set of antibodies, or they were not reported. On the other hand, it is also possible that even if the pathogenesis is the same, IPH in children fundamentally varies from IPH in adults in their disease manifestations and prognosis. The outcome in children is known to be worse than in adults [1,36].

The patients with autoantibodies in our review were older than patients without, but the difference was not statistically significant. This is likely due to the small number of patients in the antibody-positive cohort. Similarly, no significant difference has been noted in the frequency of hemoptysis, cough, dyspnea, anemia, or the "classic triad" of presentation. Although a higher number of patients in cohort B suffered from respiratory failure, this was not statistically significant due to the same reasons. Unlike pediatric patients, we did not find any evidence of worse outcomes in patients with a positive antibody regarding recurrence rate and survival.

Most of the patients included in this study were diagnosed with IPH after a consistent lung biopsy. Although most clinicians consider IPH to be an autoimmune disease [37], no histopathologic proof of this supposition has ever been obtained. There was no consistent finding of alveolar basement membrane abnormalities, immune complex deposition, or inflammatory cells invading the lung parenchyma [38,39]. The typical histopathologic description of IPH is bland pulmonary hemorrhage without evidence of vasculitis, granulomatosis, or inflammatory cellular infiltration [5,10]. Of note, several patients identified in this review who were diagnosed with IPH were in fact noted to have perivascular lymphoid aggregate chronic inflammatory cellular infiltrate on lung biopsy that was thought to be nonspecific [21,29]. We believe that findings on histopathology that are not typical of IPH should prompt physicians to search for the presence of other autoimmune diseases and have a low threshold for the evaluation of rheumatologic disorders should the suggestive symptoms arise.

Limitations of the study

Our study is not without limitations. The main weakness of this study is that only five patients were found to have autoantibodies. Although the low prevalence of autoantibodies in the adult population is a crucial finding, the low number of patients made the comparative analyses between the cohorts challenging. We only included patients reported in the literature in English, which likely reduced our overall number of patients. Finally, as all identified cases were in "case report" format, the information provided was not consistent, and there was a high chance of publication bias.

## Conclusions

The occurrence of autoantibodies is uncommon in adult IPH patients. Based on our review, only 13% of patients had a positive antibody. This is in contrast with the pediatric IPH patient population, where the prevalence is much higher, and the detected antibodies are more diverse. In this review, the patients with autoantibodies tended to be older and most of them were male, but no significant difference was found between patients with or without autoantibodies regarding their clinical manifestations. Unlike pediatric patients, adult patients with autoantibodies do not necessarily have worse outcomes.
